# Universal health care in middle-income countries: Lessons from four countries

**DOI:** 10.7189/jogh.11.16004

**Published:** 2021-11-20

**Authors:** Alexander S Preker, Daniel Cotlear, Soonman Kwon, Rifat Atun, Carlos Avila

**Affiliations:** 1 Health Investment & Financing, New York, New York, USA; 2 Palladium, Washington, D.C., USA; 3 Graduate School of Public Health, Department of Health Policy and Management, Professor and Former Dean, Seoul National University, Seoul, South Korea; 4 T.H. Chan School of Public Health, Department of Global Health and Population, Director of Global Health Systems Cluster/Professor of Global Health Systems, Harvard University, Boston, Massachusetts, USA; 5 Palladium, London, UK

## Abstract

**Background:**

In this paper, we review lessons learned about Universal Health Coverage (UHC) in middle-income countries, with specific reference to achievements and challenges observed during recent years in four middle-income to upper-middle-income countries – Mexico, Turkey, The Republic of Korea and Ukraine. Three of these countries – Mexico, the Republic of Korea, Turkey are members of the Organization for Economic Cooperation and Development (OECD). Ukraine has aspired to join Western institutions like the OECD since its independence in 1991

**Methods:**

The research included a combination of cross-sectional and longitudinal reviews of both statistical and contextual data, available from both published sources and available “grey literature” reports.

**Results:**

Based on the research, we conclude the following. First, reaching UHC is achievable in middle-income and upper-middle-income countries. It is not an unattainable goal reserved for upper income countries. Second, successes and failures are evident both in the case of countries that pursue a contributory health insurance path to UHC and those that pursue a core government funding path. Third, the devil is often in the detail. De jur*e* constitutional guarantees and national health legislation are often a necessary but do not constitute a guaranteed path to success without accompanying institutional measure to secure sustainability (political and economic) and supply and demand constraints in service provision and consumer/patient behavior. De facto, in most countries expansion in health insurance coverage does not happen “with the stroke of a pen” but require years of commitment and efforts to change the supply and demand after critical legislation has been enacted. Fourth, two major approaches dominate: incremental and “big bang” health system reforms.

**Conclusions:**

We caution against the pitfalls of over-attribution from drawing too strong conclusion from individual longitudinal country experiences (“over-determinism”) and over-generalization from broad sweeping cross-sectional statistical analysis (“reductionism”). Every country is different and needs to find its own path towards UHC considering their contextual specificities, learning from the achievements and failures of others, but not try to copy their experiences.

Faced with illness, disability and death, people throughout history, at all income levels – rich or poor – and from all cultural backgrounds have in the past and continue today to aspire to overcome their ailments and restore good health. Systems-wide changes, that allow affordable access to needed health care at the time of illness, has been a major theme for health care reform throughout the 20^th^ century. Most countries that are members of the Organization for Economic Cooperation and Development (OECD), with the exception of Mexico, Turkey and the USA have succeeded in this quest. And some countries that are not yet formally part of the OECD like the Ukraine have succeeded despite continued macro-economic challenges. [1].

Earlier contributions to this special issue of the Journal have summarized the broader principles of Universal Health Coverage (UHC), its complexity and the pitfalls of automatically equating UHC with achieving universal and affordable access to health care when needed. We will build on this story by looking at the experience of middle-income countries more broadly, and specifically at the well documented experience of Mexico [2-4], The Republic of Korea [5-7], Turkey [8-10], and the Ukraine [11-13].

Since a comprehensive review of all the different dimensions of UHC would not be possible in a short journal article, we focused our analysis on three dimensions of UHC that have appear to pose major constraints in reaching what could be considered universal access to health services at the time of illness and acceptable financial protection against its impoverishing effects, namely: (a) political and economic sustainability of the reforms: (b) demand-side constraints; and (c) supply-side constraints.

## METHODS

The research included a combination of cross-sectional and longitudinal reviews of both statistical and contextual data, available from both published sources and “grey literature” reports which were retrieved from open source data repositories, including those from the World Health Organization (WHO), the World Bank and national policy documents. We analysed “*why, how, when* and *to what effect”* policies (or lack of such policies) by governments and other groups were successful in advancing the UHC agenda or created impediments [14-19]. In particular, we analysed:

- **Political sustainability**. Reaching UHC requires a sustained effort over long periods of time and typically there are periods of lack of progress and even of reversal. We would describe the reforms, focusing on question of political sustainability in our four countries, including the links to economic sustainability.
- **Demand-side issues**. A crucial demand-side question in the last stage of UHC is how to deal with the informal sector when expanding UHC. The four countries studied have health systems that include social health insurance. In these countries there were two paths followed to cover the informal sector: The Republic of Korea and Turkey pursued a contributory path for the informal sector, developing mechanisms to raise revenues from informal sector families (in addition to providing tax subsidies). By contrast, Mexico decided not to collect revenues from the informal sector and to cover all families not included by the formal sector social security schemes into a tax-financed system. Another important demand-side challenge for these countries relates to financing and population aging.
- **Supply-side issues**. Three topics will be discussed regarding the last step in universality. The emphasis on primary health care, the policy regarding pluralism of providers; and the search for solutions to the scarcity of human resources for health.

## RESULTS

### Findings from thematic reviews

The following thematic reviews summarize progress made in Mexico, the Republic of Korea, Turkey and Ukraine in moving towards UHC during the past decades, including some of the challenges these countries face in the future in completing the UHC process.

#### Political sustainability

##### Main reform process

Mexico, the second-largest economy in Latin America, is the 15^th^ largest economy in the world, with a nominal GDP of US$1.22 trillion and a population of 120 million. **Mexico** experienced a rapid industrialization and modernization during the past century. The creation of a Social Security System in the 40s providing pensions, medical and social services was one of the major signs of progress. Private formal workers and civil servants enjoyed access to affordable and good quality services. The Minister of Health oversaw public health programs and the provision of medical services based on a national network of primary health clinics and hospitals. There was medical progress in the cities along with urbanization; there were nursing ad medical schools in every State and the National Institutes of Health were prestigious institutions. Despite this progress; the maternal and child health indicators were performing poorly and below what was expected for the economic development experienced in the 60s and 70s. The economic crisis forced the implementation of health systems reforms. There were three distinctive health reforms in Mexico; the first was an administrative reform intended to decentralize functions and devolve health facilities to the states. The second, a service provision reform targeting the rural poor by implementing a model of community engagement and mobilization. More recently, a health financing reform introduced health insurance by targeting the informal sector.

In **the Republic of Korea**, since the introduction of health insurance to the workers in large business in 1976, it was incrementally extended to workers in smaller business along with a series of pilots for the informal sector. The informal sector in rural areas joined the health insurance in 1988, and the universal coverage of population was achieved in 1989 when the informal sector in urban areas were covered. In 2000, all insurance funds were merged into a single insurance system. Since then, health reforms continued, eg, incremental expansion of benefits package, positive listing of medicines based on HTA (Health Technology Assessment), pilots of case-based payment, introduction of long-term care insurance, etc.

In 2003-2013, as part of the Health Transformation Program aimed at achieving UHC, **Turkey** introduced comprehensive changes simultaneously in health system functions of organization and governance, financing, resource management, and service delivery to address inadequate and inequitable health financing, human resources and physical capacity [8,9].

Prior to the Health Transformation Program, Turkey had a fragmented, inadequate and inequitable social insurance system. The Social Insurance Organisation for blue-collar workers who were in formal employment was established in 1945. This was followed in 1949 by the creation of the General Employees Retirement Fund for retired civil servants and their dependents and the Active Civil Servants Insurance Fund (covering civil servants in work and their dependents). In 1960, coinciding with the introduction of a new constitution, UHC became a state objective and was included in the 5-year state plans. In 1965, Active Civil Service Fund established to extend health insurance to civil servants in employment, financed by the Ministry of Finance from general budget revenues. In 1971, Bağ-Kur, the social insurance scheme for self-employed craftsmen, tradesmen, artisans, other self-employed, such as seasonal workers, and organized groups, was established. In 1980, Bağ-Kur offered health insurance to its beneficiaries. The addition of Bağ-Kur to the three other social insurance schemes meant that only citizens on low incomes and those in the informal sector who could not afford to pay for social insurance did not have coverage. In 1992, the Green Card Scheme, a non-contributory health insurance scheme for households outside the formal social insurance schemes was introduced as an interim measure until the creation of a unified health insurance scheme. However, unlike the other four insurance schemes, in its first 10 years the Green Card scheme was administered by the Ministry of Health, but without an organised insurance system and a system for means testing low-income families. In effect it functioned as a means of providing funding for uninsured individuals with low-incomes who could not meet hospital inpatient costs. Consequently, the uptake of the Green Card scheme was poor due to limited coverage of benefits – only 2.3 million people accounting for 20% of those eligible were registered under the scheme. While the Green Card Scheme provided coverage of the cost of part of inpatient care it did not so for outpatient services, medicines and diagnostic investigations. As a result, the beneficiaries incurred large amounts of out of pocket expenditures.

The health system reforms introduced as part of the Health Transformation Program in Turkey expanded health insurance and service coverage for the country as a whole, but especially for the poorest population, and provided financial risk protection for those who could not afford health care services. The rapid scale-up of health service provision and access was made possible by expanding health insurance coverage and benefits, increasing the size of the health workforce and improving its distribution through contracting, scaling up primary health care services and strengthening emergency medical services. Strategic use of the private sector by enabling the beneficiaries of health insurance (other than Green Card holders) to access private sector providers helped to rapidly scale-up health care services and to enable expanded access.

**Ukraine** is the second largest country by land size in Europe after the Russia Federation. In 2016, The Ukraine had a population of 44.4 million; 15% less than that in 1991, when the country gained independence from the Soviet Union, due to a combination of below-replacement birth rate and emigration. Key population health indicators fell after independence, they have since recovered to improve slightly compared with pre-independence levels, with more rapid improvements since 2010. Life expectancy is 64/78 for men/women (low by European standards) The probability of dying under five is 9 per 1 000 live births in 2017 (good compared with low- to middle-income countries elsewhere in the world). And probability of dying between 15 and 60 of 264/298 for men/women (which is very high by European standards). High mortality rates in the general population are mostly attributable to cardiovascular, cerebrovascular diseases and cancer.

During the 19^th^ century, **Ukraine** was a rural area largely ignored by Russia, Austria and Germany. Unlike other Central European countries like the Baltic States, Czechoslovakia, Hungary, Poland and Romania, prior to the Bolsheviks Revolution in 1917, Ukraine did not have any health insurance like the Sickness Fund System in Germany or Friendly Societies like in England. The state had little involvement in health care which was mainly a private domain between doctors and patients.

The Russian Civil War devastated Ukraine. It left hundreds of thousands homeless, and 500 000 people were killed. Many more died from the Russian famine of 1921 (primarily affecting the Russian Volga-Ural region). Nevertheless, Bolsheviks were committed to full employment and housing as well as free health care, education and social-security benefits. With the stroke of the pen, the Ukraine and other Soviet Bloc countries joined New Zealand as the first European country to introduce UHC – at least on paper.

Unfortunately, facts on the ground in the Ukraine were very different. First, most of these policies were sharply reversed by the early 1930s under the brutal regime of Joseph Stalin. Second, by the time republics like Ukraine recovered from the adverse effects of the policies under Stalin, they were quick to discover a wide range supply, demand, and performance issues in their health systems that were typical of the planned economic model of funding and production.

Ukraine, like other countries in the Union of Soviet Socialist Republics (USSR, founded in 1922) slowly introduced a totally state-run health system — the Semashko model – which was based on centralized planning and delivery of health care services, comprising an integrated and hierarchically network of government health care services for all citizens. All the health workforce became state employees. In principle, the system was intended to prioritize primary health care, with links to specialist and hospital care. It was designed to be a uniquely “coherent, cost-effective system for funding and delivering health care for the population” – precisely what many health system reformers today strive for.

In reality, it took until the 1960 for more economically advanced countries like the Baltic States, Czechoslovakia, Hungary, Poland and Romania to reach anything near universal free health care for all. And it took much longer for others in the Soviet Union like Russia, Belarus and Ukraine to reach a similar coverage, largely due to supply constraints – time to build up the infrastructure, especially in expansive rural regions and train enough doctors and nurses. And the Soviet planned economic model was not good at responding to the dynamics of supply and demand forces. It led to an overshoot with more hospital beds and staff being trained than much richer Western European countries.

Although effective during the expansion phase from the 1930 to the 1960s, by the early 1980s the Soviet Union had four times the number of doctors and hospital beds per head compared with the USA. It became both financially and politically unsustainable. As a result, the quality of medical care deteriorated well below western standards. Many medical treatments and diagnoses relied on unsophisticated, substandard and insufficient equipment. The system was plagued by shortages in and outdated drugs. Facilities had low technical standards and the health workforce had mediocre training. The physical facilities suffered from chronic low standards of hygiene, food and linen. Although, special hospitals and clinics were available for the Nomenklatura nomenklatura (Communist leadership), even these were far below Western standards.

##### Recent key reforms: when, why, what, how, and to what effect as well as associated economic sustainability/affordability and political sustainability

In **Mexico**, recent reforms included administrative overhauls intended to decentralize functions and devolve the operation and financing of health facilities and hospitals to the 32 States. In the 1980s the Mexican economy suffered its worst recession ever and the government had no reserves to repay an accumulating national debt. The WB lent under the condition that Mexico reduce its social expenditure on health and education and also moved forward with decentralization and privatization of health services. There were multiple objectives of the administrative reform including transferring fiscal responsibility to states, municipalities, and users in order to free the central government’s resources and thus repay public debt. This reform was planned to decentralize the MoH by transferring financial responsibilities to the States; however, failed in devolving also adequate decision-making authority [20]. The opposition from state governors and unions were strong and only some functions were decentralized. Labor unions opposed the decentralization and privatization of the Mexican Institute of Social Insurance (IMSS) covering the formal sector, and the decentralization was limited to the MoH in charge of the uninsured population. The reform was aimed to improve efficiency and participation. States were required to fund between 20% and 40% of health expenditure. In 1987 only 14 of the 31 states had decentralized. Several analyses showed that the decentralization reform failed to improve efficiency, increased health inequities, and had a negative impact on quality.

The next was a supply-side reform in **Mexico** that occurred in the late 80s. The context was a country with indigenous communities left behind of the country rapid urbanization and industrialization. Health services were provided in cities and the rural poor was socially excluded and underserved. There was no progress in reducing maternal, infant and child mortality and the government decided to implement inclusive social policies. The reform focused on implementing a new model of service provision with strong community engagement, social mobilization and culturally sensitive. This program operated under the presidency to coordinate all the ministries to achieve community development. The program was known as the Coordinación General del Plan Nacional de Zonas Deprimidas y Grupos Marginados (COPLAMAR). This was a federal funded program that provided high impact basic social and health services free of charge with strong emphasis on community participation. The program was the social branch of IMSS (social security for the formal sector) funded by the federal government and using the expertise, management, administrative platform, information systems, procurement and supply chain for medicines and medical products to support an efficient operation and affordable national program for rural communities (Alvarez 1989). At the center of the integrated health service provision model a physician and nurse assistant covered four communities with a population of up to 5000. Voluntary health workers, traditional healers and rural supervisors were central to this model. The model was supported by new infrastructure; 3025 medical posts and 62 rural hospitals, surgery and integrated family medicine unit. The new model increased health coverage to 10 million people living in rural and disperse communities. Community participation was key to ensure the success of the program. Improved coverage levels for family planning, vaccination, oral rehydration, treatment of pneumonia, parasitosis and tuberculosis, malaria and institutional deliveries had enormous impact. The health condition of indigenous communities was improved along with national health indicators. A reduction in outpatient visits was observed along with an increase in community health promotion and preventive activities. IMSS-COPLAMAR was able to increase coverage to 15% of the population out of the 75 million population in the 1980s and reduce maternal and infant mortality.

In 2003, a demand-side health reform was implemented in **Mexico**. It consisted in introducing a voluntary health insurance scheme targeting the poor and the informal sector. Before Seguro Popular was implemented, there were health coverage gaps mainly among the urban poor and the informal sector. The scheme also intended to provide financial protection, reducing out of pocket and catastrophic expenditure to alleviate poverty. This new reform advocated universal coverage through an insurance-based system in which the premium for the poorest people was subsidized by the government and in which public and private institutions compete to capture clients. The government strengthened its stewardship function by regulating the system, financing the services for the poor, monitoring performance and ensuring patient and consumer protection. The Law was enacted in 2004 and the objective was to provide access to an explicit package of services for people excluded from Social Security. Until the end of 2019, the scheme was covering more than 1000 health conditions including 618 surgical procedures and 670 medicines free of charge. The 2019 operating budget was reported in US$ 4 billion and the number of enrollees 53.5 million people. The scheme was voluntary, and an attractive feature of the insurance scheme was based on the premise that funding follows the patient.

A new government in **Mexico** started a counter-reform to regain centralized political and fiscal power. During the first year, the new government made legislative changes and dismantle Seguro Popular and replace it with the Mexican Institute of Health and Wellness. In February of 2020, 23 out of the 32 government states had signed framework agreements to receive resources from the federal level and coordinate the operation of the new institution. The arguments provided for the change is that the new institute will be able to “centralize the funding and increase health coverage”, enrollment is not mandatory so all citizens will have access to all health services. However, without enrollment or defined benefits, it is going to be difficult to plan for success, match the supply to the demand, and balance revenue to expenditures and money to pay for explicit health care benefits. There is limited information about operations and service delivery under the new system; however, the new institution eliminates a model where the funding follows the patient and payments are based on results. The new government reintroduced a traditional model where the MoH is in charge of financing and delivering services and funding is allocated to state health care networks regardless of the results and services provided. There is no separation of functions and the MoH is the steward, regulator and provider of services. Opposite incentives will be observed under the new funding model as compared to Seguro Popular in terms of efficiency, productivity and quality of care. Still many challenges remain in achieving integration of the system, equity, efficiencies and quality of care.

**The Republic of Korea** achieved universal coverage of population in 1989 and merged its insurance funds into a single insurer in 2000. The presidential election triggered and expedited the extension of health insurance for universal coverage of population. President Chun Doo-Whan and the presidential candidate of the ruling party, Roh Tae-Woo, were former military generals and wanted to obtain political support by proposing universal health insurance coverage [21]. The impending 1987 presidential election was the first nation-wide election with the participation of the entire population in more than 25 years. In 1986, one year before the presidential election, the Government announced plans to extend NHI to the entire self-employed.

After the NHI achieved universal coverage in 1989, the NHI system consisted of more than 350 not-for-profit insurance funds. NHI consisted of three different types of funds: health insurance for employees and their dependents; health insurance for school and government employees and their dependents; and health insurance for the self-employed. Because the ability of farmers to pay their contribution was limited, many insurance funds for the self-employed, especially those in rural areas, suffered chronic fiscal deficits. Facing the fiscal instability of self-employed funds, the government introduced a risk-sharing mechanism of cross-subsidy among insurance funds, based on catastrophic medical expense and the proportion of the elderly. However, fiscal deficit in the health funds for the self-employed continued, and there was an increasing concern over inequity among insurance funds.

The break-down of the former policy equilibrium with multiple insurance funds was driven by the first progressive government in almost 50 years [22]. With the democratization in the policy process, the new progressive government effectively mobilized farmers, the urban poor, and progressive civic groups, who were supporters of the single payer NHI system. The Republic of Korea’s civic groups were not grass-roots organizations at that time, but were led by progressive academics or active members of the former movement for democracy. Economic crisis, which resulted in the IMF rescue loan in 1997, also helped open the window of opportunity for major socio-economic policy reforms.

In **Turkey**, several factors contributed to the creation of an enabling context that made possible the transformation of the health system, including sustained leadership, political support for reforms, including from the Grand National Assembly, the Council of Ministers and most importantly from the citizens who had expectations of improved right to health and access to health care services, and effective targeting of expanded health insurance and services to the poorest segments of the population, and strategic use of available resources (financial, human and physical) to achieve scale [8,9].

In **Turkey**, the introduction of major reforms in coincided with a period of political stability when one of the political parties was able to achieve parliamentary majority, and sustained economic growth, which enabled the government to increase health expenditures at an average yearly rate of 9.1% [8,9].

In **Ukraine**, the reform process since collapse of the Soviet Union in 1991 has been slow [23].

Successive governments in the former Soviet Union and countries in Central and Eastern Europe countries, tried to overcome funding and other shortfalls of the Soviet health care system. Most turned to health insurance (both social and to a limited extent private insurance) and tried to modernize the service delivery system by reintroducing market mechanism reaching a supply and demand equilibrium, rather than rely on centralized planning and they had during the Communist era.

In this respect, **Ukraine** has been different from the rest of the former Communist countries. Although Ukraine, like many of the Soviet Republics embarked on an aggressive program of economic, political and social transformation, the rapid economic marketization and hyperinflation that followed independence caused severe economic hardship in Ukraine which also affected the health sector.

But until very recently, **Ukraine** did not put on the table a health insurance (social or private) and marketizing option for changes in funding and service. This makes the Ukraine unique as a case study because an ongoing debate in many of the other countries is that maybe it was the introduction of health insurance and market mechanisms that lead to many of their current problems rather than the legacy of the past. In the Ukraine, there have been no major fundamental reform of the system.

More recent health reforms in **Ukraine**, that began in 2010, have tried to strengthen primary and emergency care, downsize the number of hospital beds and shift from an inputs to outputs based reimbursement model. But conflict and political instability have made implementation slow and in many cases impossible. Instead, the focus has been on humanitarian priorities arising from conflict in the Crimea.

Since the transition in the 1990s, **Ukraine** has focused on decentralization in the service delivery system rather than marketizing reforms. Although decentralization has passed functional and managerial powers to the 27 regions and the local level, it has not given the lower levels of the system autonomy to take decisions over funding or the service delivery model. Restructuring of the service delivery system has been constrained by constitutional provisions that prohibit the reducing of the existing network of publicly owned health care facilities. As a result, the private sector in the Ukrainian health system has remained small, mainly of privately practicing doctors, diagnostic facilities pharmacies and dental services. Capacity planning has remained centralized and input-based, insensitive to supply and demand forces.

##### Trends in historical coverage

**Figure 1:**
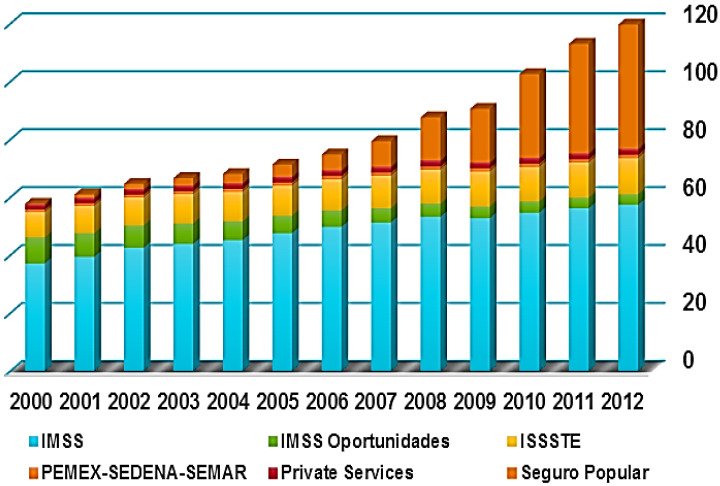
Cumulative enrollment in million population 2000-2012.

For **Mexico**, **Figure 1** presents trends in population coverage during the last 12 years. The Health System in Mexico is fragmented into several pooling funds. IMSS is the social health insurance for employees of the private sector and their dependents, there are four different financing pools for civil servants (ISSSTE), state oil owned enterprises (PEMEX), armed forces (SEDENA and SEMAR) and IMSS Oportunidades the social arm of IMSS. Seguro Popular was able to close the health coverage gap in only 10 years of operation. **Figure 1** presents the reported number of people enrolled to the different health schemes. The covered population is very close to the total population. This might be due to people enrolling to a more than one scheme under a highly fragmented health care system.

**Figure 2:**
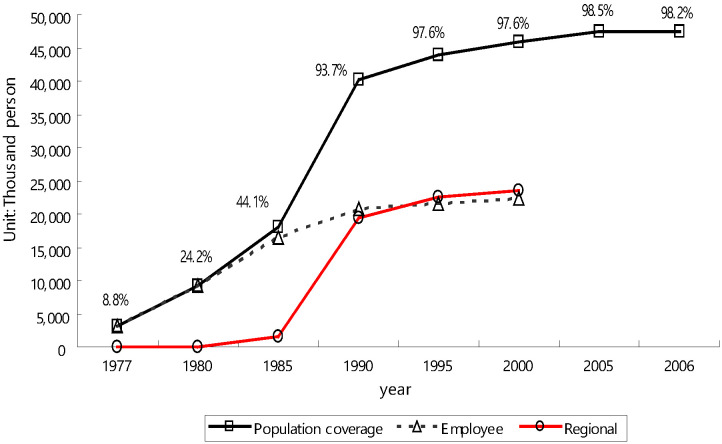
Steep increase occurred over 10-year period between 1980-1990 in Republic of Korea. Source: Kwon (2009) [21].

In the **Republic of Korea**, there has been a progressive expansion of coverage over time. From the very beginning, health insurance adopted dependents coverage, in which dependents were covered when family head joined health insurance. Historical increase in population coverage in the Republic of Korea is close to 100 percent. **Figure 2** demonstrates the period of steep increase occurred over a 10-year period between 1980 and 1990 in the Republic of Korea [24].

In **Turkey**, in 2003, 2.3 million (20% of those eligible) had registered with Green Card Scheme. By 2006, 8 · 3 million people were registered, and by 2011, when the Green Card scheme benefits package became comparable to other types of insurance, the number of enrollees increased to 9 · 1 million. To enable this expansion, the health expenditures in Turkey rose from 4.1% of the gross domestic product in 2002 to 6.1% in 2010. Between 2000 and 2010 public-sector funding increased from 63.0% of total health expenditures to 75.2%, one of the highest among middle-income countries [8,9].

The **Ukraine** reached de jure and de facto UHC in the 1960-70s. But over time, because of erosion in the quality of health care out of pocket expenditure has increased, especially since the economic reforms of the early 1990s. As a result, although on paper the Ukraine still offers UHC to its citizens in reality coverage is in terms of financial protection is much lower. Furthermore, since the transition in the 1990s government commitment to the health sector as a percentage of GDP has also dropped and out of pocket expenditure increased.

**Table 1** demonstrates the significant high out-of-pocket expenditure on health care today in Ukraine. Incomplete coverage and household spending on drugs is key source of this out-of-pocket. The scope for influencing prescribing patterns is limited because most pharmaceuticals are purchased directly by patients and also restrained by strong influence pharmaceutical companies and the liberal pharmacy dispensing procedures.

**Table 1:** Ukraine out-of-pocket expenditure on health care

	1995	2000	2005	2010	2011	2012
% public sector expenditure/total government exp.	11.4	10.2	11.9	12.6	11.8	11.5
% public sector expenditure/GDP	5.1	2.9	3.8	4.4	4.1	4.2
% Out-of-pocket expenditure/total health exp.	31.4	44.1	37.5	40.5	41.5	42.3

GDP – gross domestic product

Most recent data for Ukraine in 2016, shows that following the civil war, it has dropped and out-of-pocket expenditure increasing from its level of 42.3% in 2012, to over 50 percent (due to the recent political instability data collection and data quality is not reliable). Despite providing universal access to health care at no cost in principle, residents are therefore ending up spending significant amounts of money out-of-pocket when needing health care. And many of the problems in service delivery and supply described above remain have remained in Ukraine while improved in countries like the Baltic States, Czechoslovakia, Hungary, Poland and Romania.

In **Ukraine**, the health system continues to offer the population universal access to health care at no cost, in reality, the population bears a significant part f the burden through out-of-pocket expenditure. This leads to ineffective protection from the risk of catastrophic health care costs and the structural inefficiency, despite what may at first glance seem like a fair and progressive system of financing.

The financing of health care in **Ukraine,** both during the latter part of the Communist period and today, comes from general government revenues raised through taxation (value added taxes, business income taxes, international trade and excise taxes). Payroll taxes, insurance premiums and personal income tax is not a significant contributor to total revenues. Unlike most other countries from the former Soviet Bloc, the use of contributory social health insurance – although debated – has not been introduced in Ukraine as a source of health care financing. Out-of-pocket payments account for most other health expenditure and is a major source of hardship for both lower-income and other population groups. Voluntary private health insurance schemes, plays a marginal role, contributing less than 1 percent to total financing of the health sector. Although at first sight it may seem that this would be a progressive funding system, this is undermined by the size of the informal economy (up to 40% of GDP).

#### Demand-side issues

The history of these countries shows that the challenge in the last mile of UHC involves: coverage of the poor and of the informal sector and unification or network integration of health systems. The following sections describe these challenges and identify a rapidly developing additional challenge related to the aging of populations.

##### Sequence in the coverage of populations

In Turkey, Mexico and The Republic of Korea, the initial steps were aimed at covering wage-earners in the private formal sector and in the public sector. In Ukraine, the soviet system was declared overnight as having universal coverage; in practice it initially had better coverage for urban and better-off populations. [8,9,25]. This is different from what is taking place in Africa, where the number of schemes covering the civil servants is significantly larger than those covering the private sector.

The last mile is about covering the poor and the informal sector. In Turkey and Mexico, these populations were initially covered by public providers that provided variable quality of health care services and required high out-of-pocket payments. So, in Turkey and Mexico the poor and the informal sector already had access to these services, and the reforms to cover them with insurance took a long time and only gained strength once equity and financial protection concerns became important. In the Republic of Korea, the need to provide insurance coverage to the poor and the informal sector was more basic, as there existed very few public providers, so insurance was needed to provide access for these populations to private providers. While in Turkey and Mexico the time-lag between the creation of insurance schemes for the private formal sector and those of the poor took a long time (47 years in Turkey, more than 50 years in Mexico), in The Republic of Korea the lag was only two years.

##### Unification

All countries studied perceived unification or network integration of the health systems as part of the quest for UHC. Unification and network integration are always difficult to achieve. In these countries, “unification” is sought by developing a single payer (in other countries it is achieved through network integration– as in Germany).

**Mexico** remains fragmented. The original vision of the reform was a unified system that would cut across all of the Mexican health sector, but that idea was abandoned due to resistance from powerful labor unions at the IMSS. Also, as IMSS was perceived as being inefficient, there was reluctance to expand its role with new responsibilities. A special challenge to integration in Mexico is that IMSS (and the agency providing coverage to civil servants) are not really insurers; they own their own hospital network; hence the challenge of unifying insurance also would require the unification of the public hospital network with the hospital networks of these two institutions; hundreds of unions would oppose this.

**The Republic of Korea** managed the unification process faster than the other countries, from creation of the initial fund to unification in only 22 years. Before unification, there used to be 350 autonomous schemes. For the formal sector they were based on the workplace, for the informal sector they were based on place of residence. Unification was a reform promoted by a progressive government in year 2000. In addition to the equity objective (especially for farmers in rural areas), it also was motivated by a fiscal problem: Prior to unification, the informal sector schemes received fiscal subsidies for many years; after unification government hoped for improved fiscal sustainability through a nation-wide risk pooling.

In **Turkey** for years there was a national goal of unification –it was codified in the 1982 constitution and the 1987 basic health law—but unification only occurred three decades later, in 2011. The hardest challenge to unification was the Green Card (the health insurance for the poor). On the path to unification, its benefits were significantly improved, which lead to a large increase in enrolment and made it an important political tool. The scheme was initially managed by the MoH and the Ministry was reluctant for many years to transfer this responsibility to the Social Security Agency. As in The Republic of Korea, unification also meant an equalization of the benefit package and a tax reform that equalized contributions (as % of income) [8,9].

In the **Ukraine** unification and network integration was the foundation of the communist system of health services. The Union of Soviet Republics (USSR, founded in 1922) which slowly introduced a totally state-run health care system – the *Semashko* model – throughout the union – including the Ukraine - which was based on centralized planning and delivery of services, comprising an integrated and hierarchically network of government health care services for all citizens. All staff became state employees. In principle, the system was intended to prioritize primary care, with links to specialist and hospital care. It was designed to be a uniquely “coherent, cost-effective system for funding and delivering health care for the population” – precisely what many health care reformers today strive for. To a large extent, this system remains today, although there is now also some network integration of independent health care providers that have been established since the transition in 1990.

##### Financing of insurance or subsidies for the informal sector

Countries all over the world are making decisions about how to provide insurance or subsidies for the poor. What can be learned from these countries?

In all countries, the financing of health insurance or direct services or the poor is financed by a subsidy from government (from general taxes). What varies across countries is the way insurance coverage is financed for the informal sector or they are given direct access to free services.

In **Mexico**, Seguro Popular was originally conceived as a contributory/subsidized scheme. The draft law for SP included a premium scaled according to income class with the lowest two deciles exempt from paying; revenue collection would be a responsibility of state governments who would also receive a transfer from the Federal Government. In practice, the states decided to exempt the population from paying a premium and provided waivers to increase enrollment. The states had an incentive to do so as they received more money as transfers from the federal government for each additional enrollee they reported and there were no mechanisms for the federal government to monitor what matching was being provided by the states.

In the **Republic of Korea**, initially the government provided financial support for hospitals to open in rural areas; it also provided a partial subsidy, which was about 50% of the health insurance contributions of the self employed. Today, the self-employed pay contributions estimated on data related to income and property tax (collection is done by the NHIS). There is a controversy over the use of property value to set contributions or whether property is an adequate measure of the capacity to pay. The policy is aiming now to transition into income-based contributions for both formal sector employees and the self-employed, but the incomes would include all sources of income, including financial incomes.

In **Turkey**, the coverage of the informal sector was achieved through (i) Bağ-Kur, which enabled the extension of social insurance coverage to citizens beyond those in the formal sector and who could afford to pay for social insurance and (ii) through the Green Card Scheme, a non-contributory scheme for households with incomes below the national minimum and for families on social assistance. The poor are defined by per capita income with a simple rule: total household income/members of household less than one third of the minimum wage. For the non-poor, Turkey developed a sophisticated system to determine contributions for the self-employed. Contributions are proportional to a scoring system that depends in the taxable estimated income and on the value and size of property it occupies and on the size and age of the cars it owns.

In the **Ukraine**, the *Semashko* model is funded through budget transfer from the government not through insurance [26]. Recently, there have been a number of proposals to add insurance as a pillar to the funding system but a legal framework has not yet been passed or implemented.

##### Financing the older adults

Populations in all the countries reviewed are aging very rapidly, and older people tend to spend more in health care and very old people (usually 80+) also develop disabilities and require Long-Term-Care. Are they looking for new way to raise revenues or make payment to adapt to the trend to population aging?

In **Mexico**, there is no specific insurance scheme for long-term care, and specialized institutions for long-term care are few. Long-term care is provided either by health care providers in hospitals or at home by family members.

In the **Republic of Korea**, a Long-Term Care Insurance (LTCI) was created in 2008 to provide coverage for facility- and home-based long-term care for older people who pass the eligibility test based on functional and cognitive disability [27]. It is a separate fund from the NHI, but it is managed by the same agency (National Health Insurance Service). The contribution to LTCI is a fixed percentage of NHI contribution (currently 8.5% of NHI). It covers 9% of 65+. Since 2016, LTCI has been in deficit due to the rapid increase in eligible older people. One of the major remaining challenges is the coordination between long term care hospitals, which are funded by NHI and LTC facilities, funded by LTCI.

In **Turkey**, there is no specific insurance scheme for long-term care, and specialized institutions for long-term care are few. Long-term care is provided either by health care providers in hospitals or at home by family members.

In the **Ukraine** although the main funding for long-term care in the Ukraine is the state budget, recently an increasingly popular long-term care funding option in The Ukraine is to use mortgage contracts provided by commercial entities.

#### Supply-side issues

##### Shift of health care services from hospitals to primary health care and community

In **Mexico**, Seguro Popular (SP) was instrumental in developing a strong network of PHC providers. The essential package of services funded by SP prioritized services that were delivered at the PHC level. Between 2001 and 2011, almost 2000 ambulatory health care clinics were established, and 4000 facilities were renovated or equipped [28]. By 2012, the benefits package for SP of 284 cost-effective PHC and secondary-care interventions. Expansion of PHC services coincided with the creation of mobile health teams, health promoters, and community health coordinators to provide outreach services. The Mexican Social Security Institutions for Workers and Civil Servants also offer a comprehensive package of PHC services to their beneficiaries [27].

The **Republic of Korea** has a hospital-centric health system, with limited role for PHC. Most medical school graduates undertake residency training to become board-certified specialists, many of whom establish private clinics in the community. However, hospitals in The Republic of Korea, which have large outpatient departments, compete with physician clinics in the community. The role of PHC in The Republic of Korea may have been further undermined by the introduction of health insurance which enabled beneficiaries to access hospitals thanks to reduced financial barriers. Even though there was lower co-payment for physician clinics and higher co-pay for outpatient care in hospitals, beneficiaries preferred to access big hospitals.

In 2005, the Health Transformation Program in **Turkey** introduced a family medicine-centered PHC model, with family practices contracted to provide an expanded set of prevention services, women and childcare, mobile services, home-care for patients unable to travel to clinics, and those in nursing homes, prisons, and child-care centers [8,9,29]. By 2011, 20 000 new family medicine teams and 6250 new family centers were established covering 100% of the population [28]. Expansion of family health teams led to a 3-fold increase in utilization of PHC services per person between 2003 and 2013, immunization coverage, antenatal visits and births attended by trained health staff rose significantly with reductions in infant mortality and under-five mortality. The health systems transformation which enabled the expansion of UHC helped to increase access and utilization of health care services and satisfaction of citizens with primary health care services, which further encouraged the use of primary health care services [30]. Out-of-pocket expenditures and catastrophic expenditures more than halved [31]. User satisfaction with the health system increased from 39.5% of the population in 2003 to 75.9% in 2011 [8,9], and the expansion of the family medicine model was important in the increase in user satisfaction with the health system [28].

The evolution of the health system in **Ukraine** reflects the centrally planned normative standards for inputs with predominance of hospitals, rather than the epidemiological transitions that required strong PHC for managing chronic illness. In 2000, reforms introduced a new model for basic care based on Western concepts of PHC underpinned by family medicine principles. Family doctors/general practitioners, provide up to 60 percent of basic care, working in family medicine polyclinics or polyclinic departments. Reforms beginning in 2010, tried to prioritize PHC as a strong gatekeeper in the health system, to reduce unnecessary use of specialist services and self-referral to hospitals, but weak PHC means patients bypass PHC level to access hospitals – often using inducements and gratuities.

##### Pluralism in provision of health care services

In **Mexico**, health reforms that introduced Seguro Popular fostered public-private partnerships. While some States emphasized provision of health care services in public facilities others used strategic purchasing as an opportunity to expand engagement of private sector in provision of clinical and nonclinical services, as well as retail pharmacy services. However, enabling policies were needed to fully achieve plurality of accredited public and private providers.

The **Republic of Korea** has a pluralistic health system. The introduction/expansion of health insurance encouraged entry of new private health care providers, which currently account for more than 90% of all hospitals in the country. The national health insurance applies the same purchasing conditions (eg, fee schedules, review of medical claims, assessment of quality, no balance billing, etc.) for both public and private providers. Public hospitals tend to provide services covered by health insurance, while private hospitals also provide large volume of uninsured services, which are typically very profitable because those services are not subject to fee scheduling/regulation.

In **Turkey**, creation of pluralistic provision of health services was an explicit strategy of the HTP to use available capacity to scale up UHC, and to secure new and additional investments in the health sector. The number of private hospital sector expanded substantially after 2005 to accommodate increased patient volumes that followed strategic purchasing by Social Insurance Organisation of health care services for insurance beneficiaries from accredited private hospitals. By 2010, the Social Security Institution had contracted with 421 private hospitals (90% of large-sized private hospitals) to provide diagnostic and curative care and complex emergency services (eg, for burns intensive care, cardiovascular surgery, and neonatal care). The volume of hospital services (number of hospital visits) provided by the private sector increased from 5.7 million (4.6% of the total 124.3 million services) in 2002, to 59.1 million (17.5% of the total 337.8 million) in 2011 [8,9].

In **Ukraine**, dissatisfaction with the rigidity and low quality of the public system, have prompted patients to increasingly seek care from private health care providers (both generalists and specialist), private diagnostic centers, clinics and pharmacies. While inpatient care has remained primarily public, private ambulatory services are mostly outside the public funding streams, accounting for a large share of the out-of-pocket payments that comprise 50 percent of total health expenditures in Ukraine, with adverse consequences for equity, efficiency and financial risk protection.

##### Leverage technology and shift health care tasks from physicians to other health professionals

In **Mexico** health reforms have enabled investments in hospitals to upgrade medical equipment and in developing telemedicine networks that is used in rural areas with mixed successes.

The reforms in the Republic of Korea, Mexico, Turkey, and the Ukraine utilised technology variously.

The **Republic of Korea** has invested substantially in new health technologies for diagnosis and treatment, especially in the hospital sector, which often causes concerns for demand inducement in a private sector-dominated health system. Health insurance organization is a leading agency for IT as all providers submit claims electronically, and the insurer extensively uses ICT-based claim review and assessment, dissemination of provider performance, real-time drug utilization review (DUR), etc.

In **Turkey**, reforms coincided with substantial investment in new health technologies for prevention, diagnosis and treatment. Nationwide electronic medical records were introduced in PHC centres and in hospitals for tracking activities and for claims processing. New IT systems have enabled patients to book hospital appointments through Internet. Emergency response for medical emergencies was strengthened through the use of advanced communication, transport and medical technologies [8,9].

While **Ukraine** has extensive health infrastructure and human resources, a legacy of the Soviet Semashko health system, investments in new health technologies have been sub-optimal due to fiscal constraints.

In the four study countries, the use of tele-medicine has been muted, and task shifting of responsibilities to new cadres of health professionals limited.

#### Development of services for long-term-care sector

In Mexico, Turkey and The Ukraine, policies for establishing a long-term care insurance and sector are less well developed than The Republic of Korea. There is little formal long-term-care in **Mexico**. Households rely mainly on family networks and private sector services for a few privileged families that can afford such care.

Following the introduction of long-term care insurance in the **Republic of Korea** in 2008, the number of LTC providers rapidly expanded, from 2600 in 2009 to 5000 institutions, mostly in urban areas, while the number of home-based care agencies increased from 11 900 to 12 900 [32]. The number of care workers and nurse aides, health workers needing shorter periods of education and training than registered nurses or doctors, also increased substantially. Most of the health workforce expansion for LTC occurred in small-sized institutions (with capacity of less than 30 residents) or group homes (less than 10 residents), where market entry is relatively easy for private sector providers. The private sector comprises around 70%-80% of LTC providers.

In **Turkey** there is no specific insurance scheme for long-term care, and specialised institutions for long-term care are few. While the Ministry of Health provides home-care services, and municipalities, especially in metropolitan areas, have begun to provide services for social care, the existing scale and scope of services are not adequate to meet current and future needs. Long-term care is provided either by health care providers in hospitals or at home by family members [33].

In **Ukraine**, cash allowances are available to the elderly and providers of care for the elderly persons living alone. In 2014, there were 290 public nursing homes financed by local government providing care for more than 51 000 people (of whom 44 000 were adults). In addition, special hospital departments for elderly provide long-term care to elderly with chronic illness, while 32 hospitals provide care for war veterans and war invalids. However, the LTC capacity is not enough to meet current and future needs.

## DISCUSSION

“Rome Wasn't Built in a Day, But They Were Laying Bricks Every Hour.” (Sometimes attributed to the British Playwright, John Heywood). Likewise, in most countries expansion in health insurance coverage does not happen “with the stroke of a pen” but require years of commitment and efforts to change the supply and demand after critical legislation has been passed. Two major approaches dominate: incremental and “big bang” reforms [34]. Many countries try the “big bang” approach, passing brave progressive reforms aimed at reaching UHC quickly but end up pursuing an incremental approach during implementation.

Throughout the world, the move towards UHC has frequently been like a Tango: two steps forward and one step back. Once can apply the 20:80 rule to this process [35]. Often de jure coverage (entitlements under constitutional provisions and health care legislation) was much higher than de factor coverage (in terms of actual financial protection measured by out-of-pocket spending.

Although progress often punctuated by overlaps and setbacks, overall there has been steady progress towards expanded coverage – starting at 20 percent or less of financial protection in low-income countries and topping-out at around 80 percent. There is no country in the world that either historically or today ever achieved 100 percent de facto coverage, even if de jure they passed a “big bang” reform on paper.

**Figure 3:**
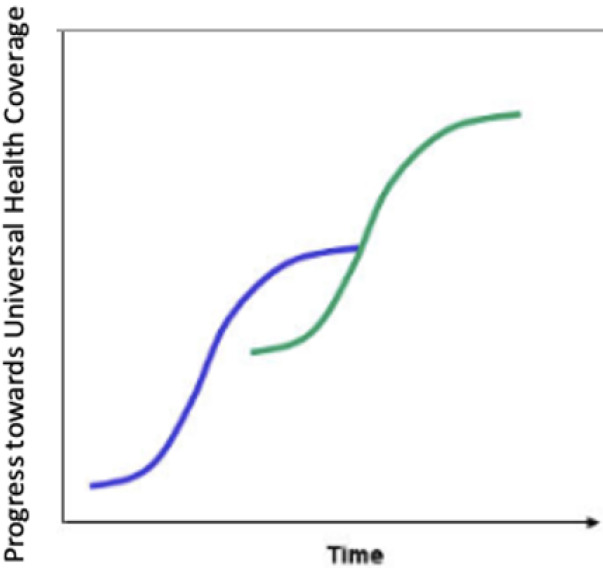
Step-wise expansion in coverage over time.

**Figure 4:**
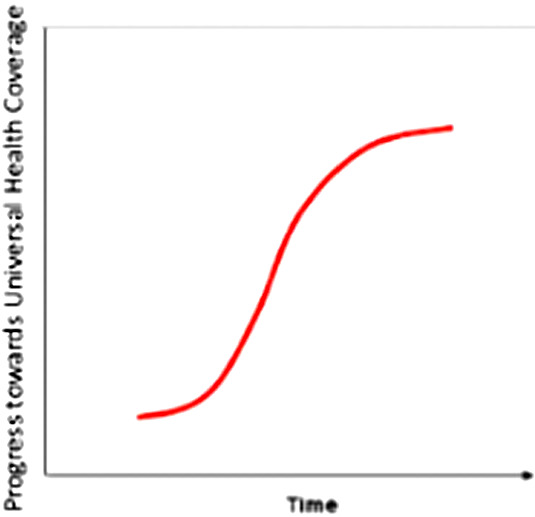
Continuous expansion in coverage over time.

All the countries we reviewed for this paper, have already passed through the steep part of the S curve and now find themselves at the upper part where progress is slower and more painful – it is much easier going from 40- 60 percent coverage than from 80 to 100 percent coverage. See **Figure 3** and **Figure 4** below shows the step-wise progress and continuously expanding coverage over time.

There are many lessons that can be learned from the experience of the four countries we have showcased in this study.

Some notable highlights that we want to emphasize include:

1. Middle income countries like Mexico, the Republic of Korea, Turkey, and Ukraine demonstrate the UHC is not an “unattainable utopian dream” but can be realized in developing not just developed countries, even if reaching 100 percent may be an elusive goal even in the OECD where most countries exclude some marginal groups (illegal immigrants, migrants, visitors, etc). Furthermore, when comparing Universal Health Coverage in the Ukraine with Mexico, the Republic of Korea and Turkey, there does not seem to be any evidence government tax funded financing provides a better road to UHC than mixed systems. See **Table 2** below for differences in UHC coverage the WHO UHC country database [36].

**Table 2:** Universal health coverage and financial protection*

	UHC service index	Population high health spending per income (SDG 3.8.2)	
	SDG 3.8.1	Greater than 25%	Greater than 10%
Mexico	76	1.9	7.1
R. Korea	80	4.0	13.5
Turkey	71	0.3	3.1
Ukraine	63	1.1	7.2

UHC – universal health coverage, SDG – Sustainable Development Goal

Source: WHO UHC country database [1].

**Figure 5:**
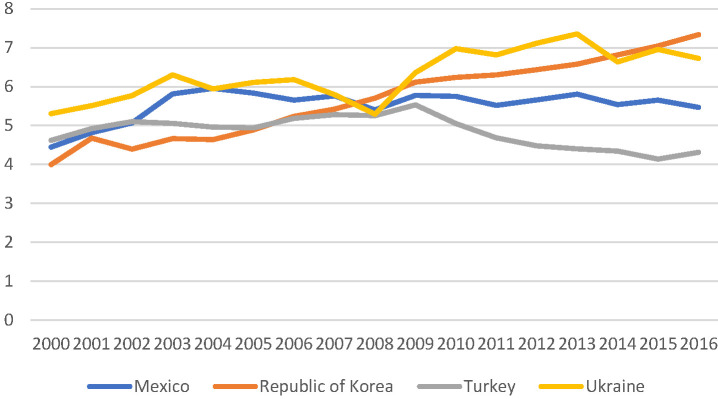
Health spending as % GDP.

**Figure 6:**
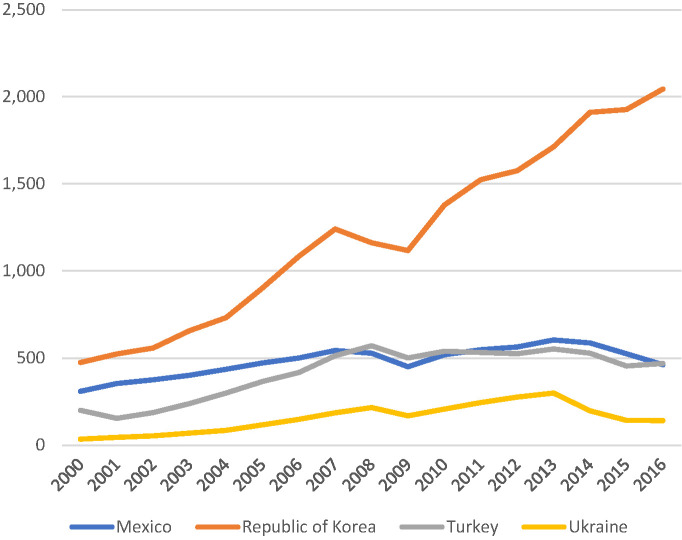
Health spending in US$ purchasing power parity (PPP). Source: Authors constructed from the WHO Global Health Expenditure Database [37].

2. The hardest period is often either at the start of the reform process and when countries are very poor and have few resources and capacity to design and implement complex reforms and at the end of the reform process when counties are richer and capacity is much higher. During the steep part of the S cure supply and demand issue related to service delivery is often a major problem (it is easier and quicker to pass a law than to build the infrastructure, capacity and demand needed to implement the reform). Later at the upper flat part of the S cure, maintaining the social cohesion needed to achieve a national consensus on redistribution and political sustainability becomes a bigger problem.

3. “Many roads lead to Rome”. Different countries choose different paths. Debate often focusses on the ideological preferences rather than substance, like insurance vs general revenue funding, freedom of choice vs compulsory social contracts and hospitals vs primary care. In reality, it may be striking a balance and doing things well (the how) that this at least if nor more important than the technical details (the what). Yet most UHC policy reforms focus precisely on the “what” not the “how”.

4. Failure to reach 100 percent coverage often has to do with – at least in part – lack of access to services especially in rural areas or lack of service mix (between primary and secondary and higher levels of care or shortages in skill mix, critical inputs or geographic penetration etc.) And at times it may be a function of demand or lack of demand for services. Providing free access to health services for everyone through various forms of subsidies does not mean that people will choose to use them. Perceptions of quality, choice of alternatives, cultural factors and many other consumer preferences may be as important in influencing demand as price.

5. Affordability is often a topic that has been used by critics to undermine UHC reforms. Level of GDP and overall wealth of a country seems to be a more significant determinant of levels of spending on health care than the policy contents like UHC. All four countries demonstrated increased spending over time as their economies grew. See **Figure 5** and **Figure 6** for trends in health spending in the four countries examined. What is striking, however is that the Ukraine has the slowest growth in spending over the period 2000-2016 in terms of spending in US$ purchasing power parity (PPP). Such under-spending by government tax funded health systems are well known. While such systems are good at imposing hard budget caps on spending they are not good at responding to growth and changing health care challenges or technical developments [38]. Furthermore, without targeting there is a tendency for subsidies to flow to the middle class and better off population groups [39]. In terms of relative spending as a percent of GDP, however, Ukraine is similar to the Republic of Korean, reflecting the difficult macro-economic times Ukraine has faced in recent years. We know that health spending is relatively inelastic relative to GDP when it comes to economic downturns, even though it is known to be elastic in growth periods [37,40-42].

The following are some of the major economic sustainability concerns often heard during health insurance reforms.

Expanding coverage did not seem to lead to greater overall health care expenditure compared with other countries. Although hard to prove and even harder to disentangle from other causal factors, none of the following commonly heard arguments against UHC seemed to be true:

- that it would be financially unsustainable to the public sector (ie, not sustainable within the existing fiscal space allocated to heath care or reasonable fiscal space expansion)
- that increase spending would have a negative impact on economic growth as a whole
- that expansion in coverage would put an upwards cost pressure on the health sector through an increased health care wage bill, increased consumption (frequency and number of users) and increased prices (ie Exp=PxV)
- that increased taxes (earmarked SS contributions or general income tax) lead to tax avoidance and increase in informal sector employment.

Based on the review, we conclude the following. First, reaching UHC is achievable in middle- and upper middle-income countries. It is not an unattainable goal reserved for upper income countries. Second, successes and failures are seen both in the case of countries that pursue a contributory health insurance path to UHC and those that pursue a core government funding path. The devil is often in the detail. *De jure* constitutional guarantees and national health legislation are often a necessary but do not guaranteed path to success without accompanying institutional measure to secure sustainability (political and economic) and supply and demand constraints in service provision and consumer/patient behavior. Finally, we caution against the pitfalls of over-attribution from drawing too strong conclusion from individual longitudinal country experiences and over-generalization from broad sweeping cross-sectional statistical analysis. Every country is different and needs to find its own path towards UHC, learning from the achievements and failures of others, but not try to copy their experiences.

We have come a long way in better understanding the complex multi-dimensional nature of UHC. More research is needed however to fully understand all the nuances of the UHC process in middle-income countries. Additional quantitative and qualitative studies are needed, both longitudinal and cross-sectional to generate evidence to better understand the introduction and scale-up of UHC, and the factors which enable or hinder UHC.

### Summary of main lessons learned

Based on the research, we conclude the following.

First, reaching Universal Health Coverage (UHC) is achievable in middle-income and upper-middle-income countries. It is not an unattainable goal reserved for upper income countries.

Second, successes and failures are evident both in the case of countries that pursue a contributory health insurance path to UHC and those that pursue a core government funding path.

Third, the devil is often in the detail, however:

- *De jure* constitutional guarantees and national health legislation are often a necessary but do not constitute a guaranteed path to success without accompanying institutional measure to secure sustainability (political and economic) and supply and demand constraints in service provision and consumer/patient behavior.
- *De facto* in most countries expansion in health insurance coverage does not happen “with the stroke of a pen” but require years of commitment and efforts to change the supply and demand after critical legislation has been enacted.

Fourth, two major approaches dominate: “*incremental”* and “*big bang*” health system reforms.

## CONCLUSION

We caution against the pitfalls of over-attribution from drawing too strong conclusion from individual longitudinal country experiences (“over-determinism”) and over-generalization from broad sweeping cross-sectional statistical analysis (“reductionism”). Every country is different and needs to find its own path towards UHC considering their contextual specificities, learning from the achievements and failures of others, but not try to copy their experiences.
